# Grand challenges in oral drug delivery

**DOI:** 10.3389/fddev.2025.1571982

**Published:** 2025-02-19

**Authors:** Driton Vllasaliu

**Affiliations:** Institute of Pharmaceutical Science, School of Cancer and Pharmaceutical Sciences, King’s College London, London, United Kingdom

**Keywords:** oral drug absorption, oral drug administration, nanomedicine, oral peptide and protein delivery, oral nucleic acids, targeted delivery

## Introduction

The oral route of medicines administration offers clear advantages. In comparison to injections, oral administration is more accessible and convenient and therefore is preferred by the patients. It also avoids potential complications and high product and healthcare costs associated with injectables, some of which require administration by healthcare professionals. Another obvious and important advantage of the oral administration route is that it can in effect achieve ‘targeted’ delivery of drugs intended for local therapeutic effect in the gut, avoiding unnecessary systemic exposure and therefore improving their safety profile.

The desirability of oral drug administration is reflected in the intensity of research efforts within this field. Many small drug molecules, almost all biologics, and a significant proportion of those in between, namely molecules within the “beyond-Rule-of-5” (bRo5) space (those characterised by a molecular weight ranging from approximately 500–1,500 Da), suffer from low or extremely low oral bioavailability. Efforts to improve the oral bioavailability of these modalities continue, as do attempts to improve targeted delivery to the gut. This article discusses some of the “grand challenges” in oral drug delivery.

## Targeted delivery to the gut

Targeted delivery to the gut remains difficult to achieve. This applies to targeted oral delivery at a macroscopic scale (i.e., different gut regions or diseased tissue) or at microscopic scale, for example, targeting specific epithelial cell subtypes. The primary strategy of stomach targeting is to achieve prolonged gastric residence time of the formulations, with gastroretentive delivery systems. However, although a large number of gastroretentive formulations have been reported, progress in clinical translation of these systems is lacking (Lou et al., 2023). Colon targeting is an often-used strategy for delivery of actives to the colon in diseases affecting this gut region, such as ulcerative colitis. However, most “colon targeted” delivery strategies exploit differences in pH between the small intestine and the colon, which often are not sufficiently prominent, particularly in disease states, hence presenting challenges for delivery. Systems making use of drug release induced through degradation of delivery systems by colonic microbiota enzymes potentially present an improved strategy, but a key challenge is to understand inter-individual variations and disease-induced changes in microbiota to ensure consistent delivery performance.

Targeting the sites of disease in the vast surface area of the gut is also highly problematic. The reported differences between pathological and healthy tissue have informed the targeting strategy. These include differences in pH, targeting of specific molecules accumulated in disease sites (e.g., positively charged proteins and reactive oxygen species) and the exploitation of the enhanced epithelial permeability (e.g., in inflammatory bowel disease) through nanomedicine (Zhang et al., 2020). However, there seems to be little progress in the clinical translation of such systems.

Being the largest endocrine organ, there is a tremendous potential to modulate the endocrine function of the gut through oral delivery systems. For example, an exciting but currently unattained prospect is to stimulate the secretion of endogenous gut hormones, including anorectic hormones in obesity or metabolic disease (Belogui, 2024). However, this would require the targeting of enteroendocrine cells, which remains an enormous challenge given that only a small proportion (around 1%) of the gut epithelial cells are of this type (Worthington et al., 2018).

Overall, the primary and currently insurmountable challenge associated with targeted oral delivery to the gut is intra- and inter-person variations in the gut physiology, which becomes even more of an issue in gut disease. To solve this challenge, a better understanding of the gut pathophysiology is required to reveal more targetable markers, in addition to developing materials with more precise responsiveness and delivery to the target cells or tissue.

## Oral delivery of bRo5 molecules

The rule of 5 (Ro5) is a set of guidelines used in drug discovery to prioritise compounds with a high likelihood of being absorbed orally. However, oral druggable space extends beyond this, to bRo5 molecules. Proteolysis-targeting chimeras (PROTACs), macrocycles and cyclic peptides are classes of modalities that fall in the bRo5 space. An interesting phenomenon observed for some bRo5 compounds is the ability to alter their conformations and molecular characteristics depending on the surrounding environment (open and polar conformations in aqueous environments versus folded and less polar conformations in nonpolar environments). There is a growing interest in the development of orally bioavailable bRo5 molecules, such as PROTACs. To achieve this, a more detailed understanding of physicochemical properties (including “chameleonicity”) that dictate absorption for these molecules is required, together with laboratory and predictive *in silico* methods for understanding and quantifying these (Price et al., 2024). This understanding is expected to inform the design of drugs in bRo5 space.

## Delivery of biologics

Oral delivery of biologics, which is otfen referred to as “a panacea in drug delivery” and keeps drug delivery researchers busy, remains an unsolved challenge. While a plethora of delivery strategies have been reported for oral biologics, those that are safe and effective *in vivo*, and with potential to translate into the clinic are much more limited. Many absorption enhancers showing efficacy *in vitro* have not progressed to the clinic and those that have seem to be limited to use with relatively low molecular weight peptides. However, larger biologic molecules require different delivery strategies. While the approval of oral semaglutide has brought hope to the field, it should be kept in mind that the success of this product has been due to a rare combination of ideal properties for oral administration, including low molecular weight, long half-life, and high potency (Aroda et al., 2022).

Nanomedicine-based approaches proposed for oral delivery of biologics are limited by the poor intestinal absorption across the intestinal barrier of most nanoparticles. The search for nanocarriers suitable for oral delivery continues, but the challenge associated with this approach will be maintaining the stability and preventing the modification of the nanoparticle delivery system in the gut environment before its interaction with the intestinal epithelium for uptake and/or absorption.

One of the most critical challenges in oral biologics delivery relates to the matching of the delivery strategy to the drug and to the clinical need. For example, while there are research efforts to enable oral delivery of monoclonal antibodies through microneedle capsules, the need and the economic viability of this approach must be questioned in light of the infrequent subcutaneous dosing of these therapies in the clinic owing to their long half-life.

When considering nucleic acid-based therapies, oral administration of these molecules for local or systemic delivery is highly desirable. One key application is oral vaccination, which is of huge benefit in achieving rapid mass immunisation during pandemic outbreaks. However, research efforts and progress in this space has been much less apparent that on oral peptides. The challenge here is the current lack of understanding whether the current technologies for nucleic acid delivery (i.e., lipid nanoparticles) may be used or may be tailored for oral administration, or whether there is a need for a completely new approach.

## Devices for oral drug delivery

Ingestible devices have been proposed not only for diagnosis of gut diseases but also oral delivery of drugs that are otherwise difficult to deliver via this route. Such ingestible devices utilise physical modes of drug delivery in the gastrointestinal tract to disrupt the mucosal barrier, for example, via direct injection, jetting, ultrasound, and iontophoresis (Byrne et al., 2021). A number of elegantly designed ingestible devices have been reported. The RaniPill capsule, which delivers the drug by transenteric injection and is the most advanced ingestible device in clinical development, has achieved bioavailability that is comparable to parenteral administration (Yamaguchi et al., 2022). While these devices may be suitable for oral delivery of biologic payloads with molecular weights extending beyond peptides, the challenge here is reproducible delivery of the payload (i.e., dose consistency), safety and cost effectiveness of the resulting therapy.

## Preclinical models for studying oral drug delivery

Preclinical models that are widely used in the study of oral drug delivery include *in silico* models to predict drug absorption, *in vitro* and *ex vivo* intestinal models and animal studies. These all have their merits and limitations. *In vitro* and *ex vivo* models are useful since, unlike animal studies, they allow researchers to carry out mechanistic studies. A key challenge is to match the model with the drug or delivery system being studied. As an example, while the widely used Caco-2 monolayer model of the human intestine may still have a place in predicting the absorption of small drug molecules and perhaps even those in the bRo5 space, the testing of larger and more complex therapeutics and delivery systems requires more complex and biologically relevant models. Recent progress in the culture of intestinal epithelial organoids, including in a configuration that suitable for oral drug delivery studies (Zhang et al., 2023), may provide a more predictive alternative to the Caco-2 system. However, this system is expensive and complex to culture, making it inaccessible to many researchers. While organ-in-a-chip technologies are interesting, the associated cost and need for specialist equipment currently make them inaccessible to most researchers. At the same time, *ex vivo* intestinal models suitable for studying oral drug absorption and delivery, including Ussing chamber systems, are becoming a rarity and the loss of such skills will have an impact on the field. There is therefore a need for inexpensive, human relevant and predictive models of the intestinal mucosa, ideally one representing the full thickness of the human intestinal mucosa, including mucus (which is not to be underestimated as a barrier), in addition to recapitulating the gut peristalsis and representing the full repertoire of intestinal epithelial subtypes. This, alongside the development of tools to study and provide an increased understanding of the gut pathophysiology, would significantly accelerate the development of oral drug delivery systems.
